# The Relationship Between Periodontal Disease and Breast Cancer: From Basic Mechanism to Clinical Management and Prevention

**DOI:** 10.3290/j.ohpd.b3904343

**Published:** 2023-02-16

**Authors:** Yuhan Zhang, Xiaolin Ren, Tao Hu, Ran Cheng, Neil A. Bhowmick

**Affiliations:** a Student, State Key Laboratory of Oral Diseases, National Clinical Research Center for Oral Diseases, Department of Preventive Dentistry, West China Hospital of Stomatology, Sichuan University, Chengdu, China. Study design, collected data, drafted and proofread the manuscript.; b Student, State Key Laboratory of Oral Diseases, National Clinical Research Center for Oral Diseases, Department of Preventive Dentistry, West China Hospital of Stomatology, Sichuan University, Chengdu, China. Study design, collected data, drafted and proofread the manuscript.; c Professor, State Key Laboratory of Oral Diseases, National Clinical Research Center for Oral Diseases, Department of Preventive Dentistry, West China Hospital of Stomatology, Sichuan University, Chengdu, China. Study conception and design, acted as scientific advisor, drafted the manuscript.; d Associate Professor, State Key Laboratory of Oral Diseases, National Clinical Research Center for Oral Diseases, Department of Preventive Dentistry, West China Hospital of Stomatology, Sichuan University, Chengdu, China. Study conception and design, acted as scientific advisor, drafted the manuscript.; e Professor, Department of Medicine, Cedars-Sinai Medical Center, Los Angeles, CA, USA. Study conception and design, acted as scientific advisor, drafted manuscript.

**Keywords:** breast cancer, clinical treatment, periodontal disease, periodontal health care

## Abstract

**Purpose:**

Periodontal disease is potentially related to certain kinds of cancer. This review aimed to summarize the relationship between periodontal disease and breast cancer, providing some strategies for the clinical treatment and periodontal health care of breast cancer patients.

**Materials and Methods:**

Systematic reviews, randomised controlled trials, prospective and retrospective clinical studies, case series and reports were collected using search terms entered into the PubMed, Google Scholar and JSTOR databases.

**Results:**

Research has provided some evidence that periodontal disease is related to the occurrence and development of breast cancer. Periodontal disease and breast cancer have some common pathogenic factors. Periodontal disease may affect the initiation and development of breast cancer involving microorganisms and inflammation. Periodontal health is affected by radiotherapy, chemotherapy, and endocrine therapy for breast cancer.

**Conclusions:**

Periodontal therapy for breast cancer patients should be performed differently according to the stage of cancer treatment. Adjuvant endocrine treatment (e.g. bisphosphonates) has a great impact on oral treatment. Periodontal therapy contributes to the primary prevention of breast cancer. Periodontal health care of breast cancer patients is worthy of clinician attention.

Periodontitis is a disruption of the normal function of the healthy subgingival biofilm with concomitant disruption of its functional properties in relation to innate defence surveillance and tissue maintenance. This leads to excessive, deregulated inflammation and tissue destruction.^13,21,60^ The results of the Chinese Fourth National Oral Health Epidemiological Survey^118^ showed that the rate of calculus among the population aged 35–44 years was 96.7%, while the rate of gingival bleeding was 87.4%. Studies have shown that periodontal disease is influenced by other diseases, e.g. diabetes, nutritional deficiencies, and obesity. Periodontitis is considered to be a contributing factor to Alzheimer’s disease and inflammatory bowel disease.^47,59,136^ Recent studies have indicated that periodontal disease promotes the occurrence of oral squamous cell carcinoma and has a potential relationship to breast cancer, oesophageal cancer, prostate cancer, haematological malignancies, and skin melanoma.^49,76^

Breast cancer is the most common cancer diagnosed in women, accounting for 30% of female cancers in 2020.^74,108^ It usually occurs in women over the age of 30, especially postmenopausal women. However, there is a good prognosis with a 5-year survival rate of 72.7%.^72^ Forman et al^38^ described breast cancer as the result of a combination of genetic and environmental factors. Race affects not only the incidence but also the degree and extent of invasion of breast cancer.^2^ The risk factors include oestrogen levels, birth history, benign breast diseases, susceptibility genes such as breast cancer susceptibility gene 1, and obesity.^62,119^ A number of recent studies have found a correlation between periodontal disease and breast cancer.^39,43,58,84^ As breast cancer may influence the treatment of periodontal diseases, the question must be addressed as to how periodontal diseases can be handled in patients with breast cancer. This review summarises the rationale for the parallels and provides some strategies for periodontal treatment and health care of patients with breast cancer.

## Materials and Methods

### Search Strategy

**Fig 1:**
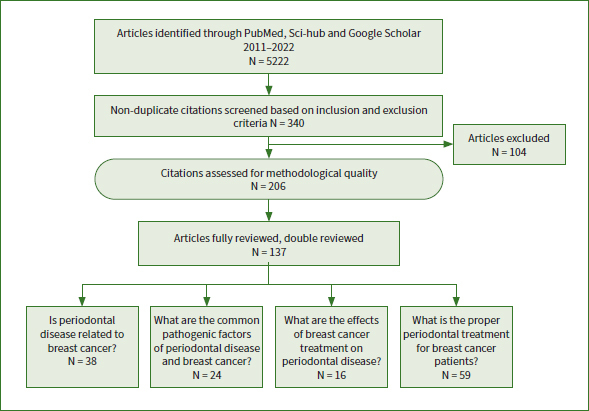
Specific search strategies and study selection.

To review the literature, we conducted electronic searches in PubMed, Sci-Hub and Google Scholar for the keywords ‘breast cancer’, ‘periodontal disease’, and ‘periodontal health care’. Specific search strategies and study selection are shown in Fig 1. First, the literature was searched to find articles that would show the incidence of breast cancer patients affected by periodontal disease. The cut-off period was from 2011 to 2022. Both English and Chinese papers were eligible. Second, representative articles from across the globe were sought that met the characteristics.

This literature search was designed to identify the following:

1. Is periodontal disease related to breast cancer?
2. What are the common pathogenic factors of periodontal disease and breast cancer?
3. What are the effects of breast cancer treatment on periodontal disease?
4. What is the proper periodontal treatment for breast cancer patients?

#### Selection of Studies

In the first screening step, two reviewers independently assessed the titles and abstracts of studies retrieved from the electronic search by using keywords. Duplicate papers obtained using different keywords were considered only once. Full-text papers were then obtained and reviewed according to the following inclusion criteria: systematic reviews, randomised controlled trials, prospective and retrospective clinical studies, case series and reports. Disagreements between reviewers were resolved by discussion. If the two reviewers did not reach a consensus, a third author was consulted.

## Results and Discussion

### Epidemiologic Evidence of a Correlation Between Periodontal Disease and Breast Cancer

Periodontal disease promotes the occurrence and development of breast cancer, especially invasive breast cancer.^58^ In a survey of 5199 subjects with a follow-up time of 7.2 years, Güven et al^43^ found that women with periodontal disease had a risk of breast cancer up to 119% higher than expected rate. A prospective cohort study of 65,869 post-menopausal women with an average follow-up of 8.32 years confirmed that elderly women with periodontal disease had a 13% increased risk of breast cancer, especially among women who had quit smoking in the past 20 years.^39,84^ Another prospective study involving 3273 subjects showed that patients with periodontal disease with molar loss had a higher risk of breast cancer than patients with normal periodontal disease.^110^ A meta-analysis^104^ showed that periodontal disease is a potential risk factor for breast cancer. Effective periodontal treatment would be an important measure to reduce the risk of breast cancer.^39^ Table 1 shows the evidence of a correlation between periodontal disease and breast cancer. The current difference in clinical findings is mainly due to the definition of periodontitis and the lack of adjustment for confounding factors in these studies.

**Table 1:** Epidemiological evidence of the correlation between periodontal disease and breast cancer

Study	Study design	Participants	Follow-up period (years)	Method	Increased risk
Güven et al^43^	Retrospective cohort study	5199	7.2	Collect diagnoses of cancer of periodontal patient	119%
Nwizu et al^84^	Prospective cohort study	65869	8.32	Oral questionnaire and cancer screening	13%
Freudenheim et al^39^	Prospective cohort study	73737	6.7	Oral questionnaire and cancer screening	26.1%
Söder et al^110^	Longitudinal prospective study	3273	16	Clinical examination, questionnaire and cancer screening	136%
Hujoel et al^54^	Prospective cohort study	6862	10	Clinical diagnosis	32%
Arora et al^6^	Prospective cohort study	8433	27	Self-report	12%
Söder et al^110^	Prospective cohort study	1586	16	Clinical diagnosis	75%
Chung et al^16^	Retrospective cohort study	42548	5	Clinical diagnosis	23%
Mai et al^77^	Prospective cohort study	1337	12.2	Measured ACH	17%
Dizdar et al^27^	Retrospect cohort study	151	12	Clinical and radiographic parameters	140%
Han et al^44^	Prospective cohort study	1979	2	Community periodontal index	56%
Sfreddo et al^103^	Case-control study	201	–	Clinical diagnosis	107%
Heikkila et al^48^	Prospective cohort study	40108	10.1	Periodontal pocket depth	19%
Michaud et al^80^	Prospective cohort study	4088	14.7	Clinical diagnosis	32%

ACH: Oral alveolar crest height.

### Aetiology

#### Bacteria and their products

**Fig 2:**
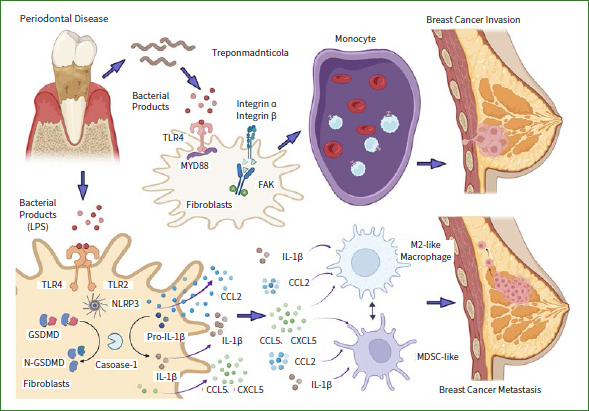
Periodontal diseases affect the invasion and metastasis of breast cancer. Periodontal pathogens mediate the crosstalk between the integrin, focal adhesion kinase and TLR4/MyD88 signalling pathways. The increased expression of TLR4/MyD88 in peripheral blood mononuclear cells promotes the invasion of breast cancer. Periodontal inflammation promotes the release of IL-1β, CCL2, CCL5, and CXCL5, recruits MDSCs to gather in the lymph nodes, and promotes the metastasis of breast cancer cells.

Oral microorganisms have the potential to colonise other organs through blood circulation. In 2014, Urbaniak et al^120^ isolated periodontal pathogenic bacteria such as *Fusobacterium* and *Streptococcus* from breast tissues of women in Canada and Ireland. Parhi et al^87^ reported that *F. nucleatum* invaded breast tumour tissue and induce tumour growth and progression, and another study suggested that the invasion of *F. nucleatum* might be induced by bacteremia.^96^ The oral and gastrointestinal microbiomes mediate steroid hormone metabolism and synthesise biologically active oestrogen mimetics that have the potential to promote the development of breast cancer.^88^ There are significant differences in the composition and proportion of breast microbial communities between breast cancer patients and healthy people. Microorganisms produce a large number of secondary metabolites that can signal mediators of breast cancer progression.^89^ For example, *Fusobacterium nucleatum* changes vascular endothelial permeability through the interaction of epithelial cadherin and adhesin junctions, promoting the expression of oncogenes and inflammatory mediators.^3,34,93,105^ Key periodontal pathogens, such as *Porphyromonas gingivalis, Treponema dentata* and *Fusobacterium nucleatum,* promote cancer invasion through the crosstalk between integrin and TLR4/MyD88 signalling pathways. The expression level of TLR4/MyD88 is positively related to breast cancer-cell metastasis (Fig 2).^61,132^

#### Local and systemic inflammation

Periodontal inflammation has a systemic effect. Researchers have hypothesised that bacterial-induced inflammation disrupts the orderly progression of stem-cell hierarchy and has a role in the pathogenesis of breast cancer; although unproven, this hypothesis is supported by the available evidence.^78^ Cheng et al^12^ found that periodontal inflammation promotes metastasis of breast cancer. Pyroptosis induces IL-1β and downstream signals CCL2, CCL5, and CXCL5.^12^ The chemokines recruit myeloid-derived suppressor cells (MDSCs) and macrophages, finally promoting the generation of a pre-metastatic niche in the inflammatory site.^11^ Polymorphonuclear neutrophils not only result in tissue destruction and bone resorption, but also induce systemic effects that may contribute to the interaction between periodontal disease and other inflammatory conditions.^36^ Chronic inflammation influences the initiation, development and behaviour of breast cancer (Fig 2).^20^ C-reactive protein (CRP) is a classic marker of acute and chronic inflammation. Elevated plasma CRP levels increase the risk of breast cancer.^42^ In patients with periodontal disease, increased plasma CRP levels may affect the occurrence and development of cancer.^8^ The core transcription factor RANK and its ligand RANKL also increase in patients with periodontal disease, and these factors may play an important role in the occurrence and metabolism of breast cancer.^18,133^

Efficient daily oral health care and periodontal treatment can reduce dental plaque accumulation and reduce the impact of microbial factors on the initiation and development of breast cancer. Early detection, diagnosis and treatment of periodontal disease can minimise the impact of inflammatory factors.

#### Host genetics

Various studies have found that periodontal disease and breast cancer have common pathogenic factors. The loss of α-v-β-6 integrin function causes periodontal disease and is also related to the occurrence of breast cancer.^67^ According to bioinformatics analysis, serpin family A member 1 (SERPINA1) and transferrin (TF) are common target genes of periodontitis and breast cancer.^75^

### Common Pathogenic Factors

Oestrogen is closely related to periodontal health and the occurrence of breast cancer. Abnormal function of oestrogen and its receptor (ER) affects the resorption of alveolar bone and the differentiation of periodontal ligament stem cells, thereby affecting the process of periodontal disease. It also participates in the occurrence and development of breast cancer.^73,79^ Excessive accumulation of oestrogen in menopausal women increases the risk of breast cancer. The type and proportion of ER expressed by breast cancer patients changes with disease progression. Oestrogen receptors are divided into two types: α and β. The expression of ER and the ERα:ERβ ratio can predict the prognosis of breast cancer patients.^91^ ERα and Erβ in periodontal ligament cells also affect the health of periodontal tissues. Oestrogen has different effects on the oral cavity of women during different parts of the reproductive life-cycle.^83^ Pregnancy gingivitis is related to oestrogen changes: the oestrogen increase during pregnancy affects the composition and proportion of the periodontal oral microbiome.^5^ Oestrogen promotes gingival inflammation and the accumulation of inflammatory cells in periodontal tissue, impacting bone resorption by mediating osteoclastic activity.^100,125^ Decreased oestrogen levels after menopause also exacerbate osteoporosis and alveolar bone resorption.^95^ Taichman et al^117^ indicated that postmenopausal women who did not receive oestrogen supplementation had twice the risk of losing alveolar bone as post-menopausal women who received supplementation; this risk is three times higher than that of pre-menopausal women.^117^ Compared with nonusers, post-menopausal women who use hormone replacement therapy have a 24% lower risk of tooth loss.^106^

For patients who have great changes in oestrogen levels, such as women during pregnancy and menopause, more attention should be given to oral health. Due to the relationship between periodontal disease and breast cancer, genetic screening of groups at high risk for the two diseases provides new possibilities.

### Clinical Therapy

Breast cancer is divided into 5 molecular subtypes based on human epidermal factor growth receptor 2 (HER2) and hormone receptors. Different molecular subtypes have different treatment methods.^129^ Treatment of breast cancer includes radiotherapy, chemotherapy, surgical treatment, endocrine therapy, and targeted therapy. Of these, endocrine therapy, radiotherapy and chemotherapy have an impact on oral health.^52^

#### Endocrine therapy

Patients with HER2+ breast cancer should be provided with adjuvant endocrine therapy for at least 5 years after surgery. Adjuvant endocrine therapy for breast cancer reduces oestrogen levels and affects the development of periodontal disease. Low levels of oestrogen reduce bone density, leading to osteoporosis and promoting alveolar bone loss.^64^

Pre-menopausal patients are often recommended to use tamoxifen, a nonsteroidal triphenylethylene derivate that blocks the actions of oestrogen.^1,15^ Aromatase inhibitors that stop the production of oestrogen are recommended for post-menopausal patients as adjunctive therapy.^15^ Tamoxifen and aromatase inhibitors have different effects on the periodontal status of breast cancer patients. Tamoxifen has less impact on periodontal status than aromatase inhibitors.^121^ Human gingival fibroblasts are a target tissue for sex hormones, and in-vitro studies have demonstrated that tamoxifen may decrease the stimulatory effect of oestrogen on human gingival fibroblast proliferation.^121^ Aromatase inhibitors increase periodontal probing depth, dental plaque accumulation, attachment loss and alveolar bone loss. Calcium supplementation partly alleviates the extent.^11,115^ Anti-oestrogen therapy affects personal mental status, including fatigue and depression, resulting in health care neglect.^28^

To block the osteoclastic activity of low levels of oestrogen, bisphosphonates are often given to breast cancer subjects with a risk of bone metastasis. However, bisphosphonates are definitely related to the development of osteonecrosis of the jaw. Clinical trials demonstrated that lowering the dose of bisphosphonates did not negatively impact breast cancer metastasis.^35,41^ Such bone-targeted therapy adjustments may benefit oral health.

#### Radiotherapy and chemotherapy

Radiotherapy and chemotherapy cause damage both to cancer and normal cells. Common oral complications of radiotherapy include oral mucositis, xerostomia, and oral bacterial infections.^101^ Chemotherapy leads to cytostatic and cytotoxic effects. Oral tissues are sensitive to chemotherapy and are prone to oral mucosal inflammation.^45^ Studies have shown increased complexity of oral bacterial profiles in patients receiving cancer chemotherapy.^81^ Vozza et al^128^ reported that the incidence of periodontal diseases in patients with malignant solid tumours reached 35.2%. Poor oral hygiene makes oral microbiome shift towards the more pathological end of the spectrum, increasing the risk of oral infection and the long-term quality of life of breast cancer patients.

### Periodontal Health Care for Breast Cancer Patients

Periodontitis affects the quality of life of breast cancer patients. The Oral Care Guidelines of the National Institute of Dentistry and Craniofacial Institute of the USA proposed that, although priority is often given to the treatment of cancer, focus should also be directed at prevention and amelioration of complications that may occur as a result of the disease and/or its treatment.^131^ In this respect, oncologists and attending physicians may refer patients to dentists. However, most dentists lack relevant knowledge. A survey in Michigan, USA, found that more than 70% of dental hygienists are not familiar with clinical treatment guidelines for breast cancer. More than 90% of dental hygienists do not understand the mechanism of breast cancer-related treatment and the risk of damage to periodontal tissue during the entire breast cancer treatment process.^114^ A personalised periodontal treatment plan based on the general condition, oral condition and treatment tolerance of breast cancer patients is suggested.

**Fig 3:**
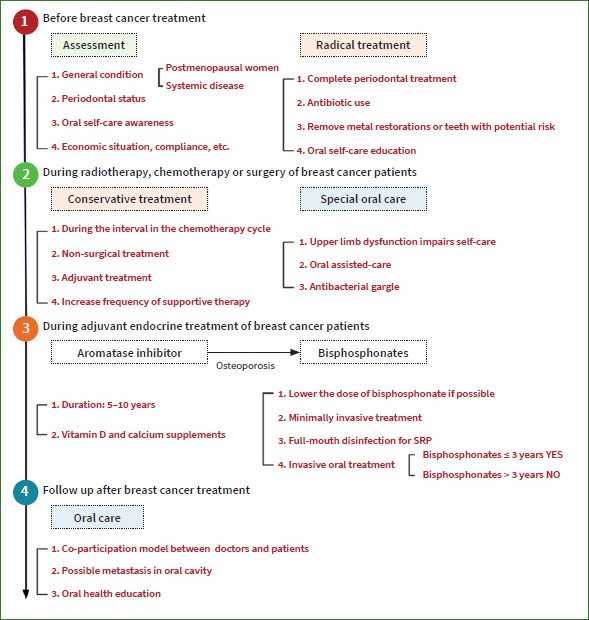
Periodontal health care for breast cancer patients is carried out in stages according to the treatment stage. 1. Periodontal care before breast cancer treatment; 2. Periodontal care during radiotherapy, chemotherapy and surgery; 3. Periodontal care during adjuvant endocrine treatment of breast cancer patients; 4. Follow-up after breast cancer treatment.

In brief, periodontal therapy for breast cancer patients should be performed in stages: before cancer treatment, during cancer treatment and during adjuvant endocrine treatment (Fig 3).

### Periodontal Care Before Breast Cancer Treatment

A case-control study of post-menopausal, elderly breast-cancer patients found that the prevalence of periodontal disease was 98% in breast cancer survivors.^4^ It has been reported that nonsurgical periodontal treatment before chemotherapy significantly reduces periodontal inflammation markers in gingival crevicular fluid.^126^ Therefore, evaluating periodontal health and optimising oral supportive care appear essential to ensure appropriate management in breast cancer-treated patients.^22^

#### Comprehensive assessment

Comprehensive assessment includes the patient’s general condition, periodontal condition, and oral self-care awareness.^30,94^ The treatment plan for breast cancer should also be taken into consideration.

##### 1. General conditions

General conditions include sex, age, menstrual history, general physical condition, other basic diseases, and tumour location and stage. Breast cancer is more common in post-menopausal women, who may suffer from dry mouth or accelerated periodontal attachment loss.^55^ Sex, age, and menstrual history have a great impact on oral health.

Screening should be performed for systemic diseases, especially those associated with periodontal diseases, e.g. diabetes, hypertension, coronary heart disease, liver disease, Alzheimer’s disease, and rheumatoid arthritis.^63^

##### 2. Periodontal status

Periodontal status includes probing depth, attachment loss level, bleeding on probing, tooth mobility, alveolar bone resorption, bifurcation lesions, and occlusal conditions. As an independent risk factor for the deterioration of periodontal disease, alveolar bone resorption greater than 1/3 should be evaluated for a personalised plan.^111^

##### 3. Assessment of oral self-care awareness

An individual’s awareness of oral health measures influences the likelihood of seeking oral care to achieve optimal oral health status. Oral self-care awareness assessment is the top priority, and patients’ willingness and ability to perform oral self-care determine the ultimate efficacy of periodontal treatment before cancer treatment.^46^

##### 4. Other assessments

Clinicians should evaluate the patient’s economic situation and compliance, for example, in choosing an appropriate treatment plan.

#### Periodontal treatment

##### 1. Radical periodontal treatment

The process of periodontal treatment for breast cancer patients involves four phases: initial therapy, periodontal surgery, restorative therapy, and supportive care.^69^ Periodontal therapy for breast cancer patients should be arranged before radiotherapy or chemotherapy.^134^ Radiotherapy and chemotherapy can only be performed at least two weeks after periodontal surgery when soft tissue meets the standard of healing.^10^ Patients with breast cancer may use antibiotics as appropriate during treatment of periodontal disease to prevent infection.^86^ For example, during the chemotherapy period, while checking the white counts, antibiotic coverage may be considered when neutrophil counts are less than 500 cells/ml, if the treatment cannot be delayed until counts exceed 1000 cells/ml.^31^ Ultrasonic instruments remove less root structure than hand instruments, but leave behind a rougher surface. Hand instrumentation has been recommended to smooth the root surface after ultrasonic use as a final finishing procedure in the treatment of periodontitis-affected roots.^68^

##### 2. Oral self-care

Patients with breast cancer should pay more attention to oral self-care. Dentists should make suggestions for choosing proper oral hygiene devices (toothbrushes, dental floss, rinsing devices and gargles) and proper oral hygiene methods. Currently, the Bass or modified Bass toothbrushing method at least twice a day is usually recommended.^26^ The public's awareness regarding the use of dental floss, dental irrigators, and interproximal brushes as preventive oral health-care behaviour needs to be raised.^53,90^

##### 3. Other preparations

Metal dental restorations will have an impact on the radiotherapy dose. Metal materials should be avoided to reduce the impact on subsequent treatment.^40^ Poorly designed restorations also have a negative effect on the oral health-related quality of life of breast cancer survivors.^57^ Teeth that may pose a future problem should be extracted as well. A nontraumatic extraction method is recommended for patients who will take bisphosphonates. Some orthodontic elastic devices are used to achieve complete tension-free closure of the wound to reduce the possibility of necrosis of secondary bone.^24,50,97^

### Periodontal Care of Breast Cancer Patients During Radiotherapy, Chemotherapy or Surgery

#### 1. Conservative periodontal treatment

Periodontal treatment during breast cancer therapy should be performed with caution. The cytotoxicity of chemotherapy drugs can suppress bone marrow and reduce white blood cell counts, thus affecting the functioning of the immune system.^37^ Periodontal treatment should be performed before white blood cells decrease. Appropriate dental and periodontal treatment should be given 2 to 3 weeks after chemotherapy or before the next chemotherapy.^31^ Some necessary treatment can be carried out during the interval in the chemotherapy cycle. Studies have shown that nonsurgical periodontal treatment is less effective for breast cancer patients undergoing chemotherapy than for ordinary periodontal patients. However, 6 months after nonsurgical treatment, the level of alveolar bone in breast cancer patients can also be significantly improved.^7,123^ The risk of oral infection should be fully considered in this period. However, periodontal treatment can still be performed under sufficient supervision. Adjuvant treatments can be added, and the treatment schedule can be extended. Oral hygiene also needs to be checked regularly during treatments such as radiation and chemotherapy.^19,32^ Due to the increased risk of periodontal disease in breast cancer patients, patients should accordingly increase the frequency of supportive periodontal therapy.

#### 2. Oral self- and assisted-care

Treatment of breast cancer is often accompanied by complications. In the first 1–5 weeks of radiotherapy, 90% of patients have acute complications on the skin of the axillary, groin, and other treatment areas, manifested as itching, redness, dryness, blisters, ulcers and even necrosis.^98^ Axillary lymph node dissection of breast cancer patients may cause upper-limb lymph node oedema, interfere with upper-limb blood circulation, and even cause upper-limb dysfunction.^25,124^ Studies found that 55.4% of women had various degrees of upper-limb dysfunction, which may be related to factors such as lymphadenectomy, lymphedema, presence of pain, and intercostobrachial nerve injury.^109^ Muscle damage and pain around the surgical and radiation fields are related to upper-limb dysfunction in breast cancer patients after long-term treatment.^9,23^ Because of the pain and discomfort caused by upper-limb dysfunction, patients may resist conventional oral health care methods. Therefore, clinicians should pay attention to the oral hygiene of breast cancer patients during treatment. If necessary, daily oral care should be provided by family members or nurse attendants.

The risk of oral infection increases during chemotherapy. A survey found that the oral infection rate reached 17.39% during chemotherapy.^70^ In addition to toothbrushing and interproximal cleaning, patients are advised to use an antibacterial gargle 3–4 times a day, such as chlorhexidine. If infection occurs, patients should maintain oral hygiene and receive antibiotic treatment or supportive treatment.^130^ However, most dentists provide insufficient attention to the oral health care of breast cancer patients during cancer treatment.^116^ This suggests that dentists should be more aware of the importance of oral health care to breast cancer patients.

### Periodontal Care of Breast Cancer Patients During Adjuvant Endocrine Treatment

#### 1. Periodontal care for endocrine medicine

The National Comprehensive Cancer Network guidelines recommend adjuvant endocrine therapy for at least 5 years after surgery, radiotherapy or chemotherapy. For high-risk patients, this can even extend to 10 years.^127^ Tamoxifen, ovarian function suppressors and aromatase inhibitors are usually used.^14^

Among the above-mentioned medications, aromatase inhibitors may aggravate the progression and bone loss of periodontitis. Calcium supplementation partly alleviates the extent.^11^ Breast cancer patients using aromatase inhibitors can take a weekly dose of 10,000 IU of vitamin D and a daily calcium supplement of 1000 mg to prevent bone loss and periodontal attachment loss.^99^

#### 2. Periodontal care for bisphosphonates

Lower oestrogen levels induced by endocrine treatment reduce bone density, leading to osteoporosis. Bone-mineral density scores (T-scores) reflect the relative differences in bone-mineral density between subjects and young adults.^135^ Patients with a T-score less than -2.5 are recommended to receive an intravenous administration of bisphosphonates for osteoporosis.^56^ During the period of endocrine therapy, especially for patients taking bisphosphonates, oral and periodontal treatment should be performed carefully. Bisphosphonate-related osteonecrosis of the jaw (BRONJ) is the presence of exposed bone in the maxillofacial region over 8 weeks with a history of bisphosphonates.85 Local infections were shown to precede the onset of ONJ in the majority of cases.^71^ As recommended by expert panels, prevention of ONJ should start before patients receive bisphosphonates.^17,65^ Among the risk factors for BRONJ, tooth extraction accounts for 35.1%, and periodontitis accounts for approximately 24.6%.^66^

As mentioned before, a lower dose of bisphosphonate will benefit oral health. Moreover, periodontal treatment should be minimally invasive to minimise the possibility of invasive operations being complicated by osteonecrosis. It is recommended that dentists use the full-mouth disinfection (FMD) technique.^29^ The goal of FMD is to minimise the risk of recontamination by performing full-mouth SRP within 24 h, in combination with comprehensive disinfection of all oropharyngeal niches using chlorhexidine during mechanical treatment and up to 2 months thereafter.^113^ FMD has modest additional clinical benefits over subgingival scaling and root planing (Q-SRP).^33^ If invasive periodontal treatment (such as surgery) is needed, the period of time that the patient takes bisphosphonates should be considered. If this is less than 3 years, surgical treatments such as extractions, apicectomies, and periodontal scaling treatments can be performed without risk. With bisphosphonate treatment lasting more than 3 years, it is advisable to avoid extractions and manipulation of bone tissue.^82^ There is no evidence that temporarily stopping bisphosphonates can reduce the risk of periodontal surgery.

Currently, there is no recognised and effective treatment for osteonecrosis caused by bisphosphonates. A case report has shown that BRONJ around osseointegrated implants was cured by using antimicrobial, regenerative and biostimulatory therapies.^122^ A similar approach can also be used for periodontal therapy.

### Follow-up after Breast Cancer Treatment

Oral care after breast cancer treatment should adopt the co-participation model between doctors and patients, who ideally interact with each other on the basis of sufficient respect. The patients provide information about the diseases, while the doctor provides feedback on the treatment plans and potential risks after obtaining the patient’s consent. Patients actively participate in the implementation process of the treatment plan and provide information routinely so that the accuracy and effectiveness of medical activities can be improved.^137^

Dentists should also pay attention to oral manifestations. Metastatic breast cancer may be accompanied by symptoms of periodontal abscess.^92^ Oral health education is necessary even after breast cancer treatment. Studies have shown that breast cancer patients after outpatient treatment and during follow-up have a high demand for cancer and related care information.^102,107^ Controlled trials have shown that breast cancer patients who have received health education have a better quality of life after surgery.^51^ When breast cancer patients undergo routine re-examinations for malignant tumours, they should also undergo regular oral examinations.

## Conclusion

As an oral inflammatory disease, periodontal disease affects multiple systems of the body. At present, the relationship between periodontal disease and breast cancer is supported by strong evidence. Periodontal disease may affect the initiation and development of breast cancer in many ways, for instance, via microorganisms and inflammation. Due to the complicated aetiology of periodontal disease and the heterogeneity of cancer, more specific mechanisms remain to be explored.

For post-menopausal patients with periodontal disease, periodontal treatment may aid in primary prevention of breast cancer. It may also reduce the risk of breast cancer influenced by periodontal disease. Dentists should consider the systemic impact of cancer treatment options on breast cancer patients and carry out oral interventions in a timely manner to limit suffering. Attention should also be paid to personalised oral care for breast cancer patients. Periodontal health care should be carried out in stages according to the cancer treatment process to improve the quality of life of patients. With developments from basic research, clinical practice may be improved in the future. In addition to clinical treatment, more emphasis should be given to prevention before cancer initiation and progression.

## References

[ref1] Abdullah H, Mamat N, Zakaria NM, Mohd Yunan N, Noor Hisham M, Hapidin H (2021). Combination effect of Tamoxifen and ascorbic acid treatment on breast cancer cells (MCF-7) and cervical cancer cells (HeLa). Malay J Health Sci [Jurnal Sains Kesihatan Malaysia].

[ref2] Allan K, Berger NA, Li L, Thompson CL (2017). Short sleep duration as a contributor to racial disparities in breast cancer tumor grade. Family Med Commun Health.

[ref3] Alon-Maimon T, Mandelboim O, Bachrach G (2022). Fusobacterium nucleatum and cancer. Periodontol 2000.

[ref4] Amódio J, Palioto DB, Carrara HH, Tiezzi DG, Andrade JM, Reis FJ (2014). Oral health after breast cancer treatment in postmenopausal women. Clinics (Sao Paulo).

[ref5] Apoorva SM, Suchetha A (2010). Effect of sex hormones on the periodontium. Indian J Dent Sci.

[ref6] Arora M, Weuve J, Fall K, Pedersen NL, Mucci LA (2010). An exploration of shared genetic risk factors between periodontal disease and cancers: a prospective co-twin study. Am J Epidemiol.

[ref7] Assis R, Silva-Junior M F, Santos M, Pereira TCR, Feitosa ACR, Azevedo-Vaz SL (2016). Radiographic bone evaluation after periodontal full mouth disinfection treatment in women undergoing chemotherapy or hormone therapy with Tamoxifen. Pesq Brasil Odontopediatr Clín Integ.

[ref8] Bansal T, Pandey A, Deepa D, Asthana AK (2014). C-reactive protein (CRP) and its association with periodontal disease: a brief review. J Clin Diagn Res.

[ref9] Brookham RL, Cudlip AC, Dickerson CR (2018). Quantification of upper limb electromyographic measures and dysfunction of breast cancer survivors during performance of functional dynamic tasks. Clin Biomech (Bristol, Avon).

[ref10] Bueno AC, Ferreira RC, Barbosa FI, Jham BC, Magalhães CS, Moreira AN (2013). Periodontal care in patients undergoing radiotherapy for head and neck cancer. Support Care Cancer.

[ref11] Cepa M, Vaz C (2015). Management of bone loss in postmenopausal breast cancer patients treated with aromatase inhibitors. Acta Reumatol Port.

[ref12] Cheng R, Billet S, Liu C, Haldar S, Choudhury D, Tripathi M (2020). Periodontal inflammation recruits distant metastatic breast cancer cells by increasing myeloid-derived suppressor cells. Oncogene.

[ref13] Cheng R, Hu T, Bhowmick NA (2015). Be resistant to apoptosis: a host factor from gingival fibroblasts. Cell Death Dis.

[ref14] Chinese Anti-Cancer Association Breast Cancer Professional Committee (2021). Chinese Anti-Cancer Association Breast Cancer Diagnosis and Treatment Guidelines and Standards (2021 Edition). Chinese J Cancer.

[ref15] Chinese Expert Consensus on Endocrine Therapy of Breast Cancer Expert Group (2015). Chinese Expert Consensus on Endocrine Therapy of Breast Cancer (2015 Edition). Chinese J Cancer.

[ref16] Chung SD, Tsai MC, Huang CC, Kao LT, Chen CH (2016). A population-based study on the associations between chronic periodontitis and the risk of cancer. Int J Clin Oncol.

[ref17] Colella G, Campisi G, Fusco V (2009). American Association of Oral and Maxillofacial Surgeons position paper: Bisphosphonate-Related Osteonecrosis of the Jaws – 2009 update: the need to refine the BRONJ definition. J Oral Maxillofac Surg.

[ref18] Czupkallo L, Rahnama M, Kielbowicz D, Lobacz M, Kozicka-Czupkallo M (2016). Bone metabolism and RANKL/RANK/OPG trail in periodontal disease. Curr Iss Pharm Med Sci.

[ref19] D Dharan SR, Jagannathan N (2015). Oral complications due to radiotherapy and chemotherapy in cancer patients. Dent Med Prob.

[ref20] Danforth DN (2021). The role of chronic inflammation in the development of breast cancer. Cancers (Basel).

[ref21] Darveau RP, Curtis MA (2021). Oral biofilms revisited: A novel host tissue of bacteriological origin. Periodontol 2000.

[ref22] de Bataille C, Castellan M, Massabeau C, Jouve E, Lacaze JL, Sibaud V (2021). Oral mucosal changes induced by adjuvant endocrine therapies in breast cancer patients: clinical aspects and proposal for management. Support Care Cancer.

[ref23] De Groef A, Meeus M, De Vrieze T, Vos L, Van Kampen M, Christiaens MR (2017). Pain characteristics as important contributing factors to upper limb dysfunctions in breast cancer survivors at long term. Musculoskelet Sci Pract.

[ref24] Decker AM, Taichman LS, D’Silva NJ, Taichman RS (2018). Periodontal treatment in cancer patients: an interdisciplinary approach. Curr Oral Health Rep.

[ref25] Devoogdt N, Van Kampen M, Christiaens MR, Troosters T, Piot W, Beets N (2011). Short- and long-term recovery of upper limb function after axillary lymph node dissection. Eur J Cancer Care (Engl).

[ref26] Ding Y, Yang MB, Wu YF (2002). Prevention of periodontal disease-a mechanical method for self-plaque control. J Dent Periodontol.

[ref27] Dizdar O, Hayran M, Guven DC, Yılmaz TB, Taheri S, Akman AC, Bilgin E, Hüseyin B, Berker E (2017). Increased cancer risk in patients with periodontitis. Curr Med Res Opin.

[ref28] Eagle I, Benavides E, Eber R, Needleman I, Worthington HV (2016). Periodontal health in breast cancer patients on aromatase inhibitors versus postmenopausal controls: a longitudinal analysis. J Clin Periodontol.

[ref29] Eberhard J, Jepsen S, Jervøe-Storm PM, Needleman I, Worthington HV (2008). Full-mouth disinfection for the treatment of adult chronic periodontitis. Cochrane Database Syst Rev.

[ref30] Eke PI, Page RC, Wei L, Thornton-Evans G, Genco RJ (2012). Update of the case definitions for population-based surveillance of periodontitis. J Periodontol.

[ref31] Epstein JB, Stevenson-Moore P (2001). Periodontal disease and periodontal management in patients with cancer. Oral Oncol.

[ref32] Epstein JB, Thariat J, Bensadoun RJ, Barasch A, Murphy BA, Kolnick L (2012). Oral complications of cancer and cancer therapy: from cancer treatment to survivorship. CA Cancer J Clin.

[ref33] Fang H, Han M, Li QL, Cao CY, Xia R, Zhang ZH (2016). Comparison of full-mouth disinfection and quadrant-wise scaling in the treatment of adult chronic periodontitis: a systematic review and meta-analysis. J Periodontal Res.

[ref34] Fardini Y, Wang X, Témoin S, Nithianantham S, Lee D, Shoham M (2011). Fusobacterium nucleatum adhesin FadA binds vascular endothelial cadherin and alters endothelial integrity. Mol Microbiol.

[ref35] Fehm T, Felsenberg D, Krimmel M, Solomayer E, Wallwiener D, Hadjii P (2009). Bisphosphonate-associated osteonecrosis of the jaw in breast cancer patients: recommendations for prevention and treatment. Breast.

[ref36] Fine N, Chadwick JW, Sun C, Parbhakar KK, Khoury N, Barbour A (2021). Periodontal inflammation primes the systemic innate immune response. J Dent Res.

[ref37] Fontanella C, Bolzonello S, Lederer B, Aprile G (2014). Management of breast cancer patients with chemotherapy-induced neutropenia or febrile neutropenia. Breast Care (Basel).

[ref38] Forman MR, Winn DM, Collman GW, Rizzo J, Birnbaum LS (2015). Environmental exposures, breast development and cancer risk: Through the looking glass of breast cancer prevention. Reprod Toxicol.

[ref39] Freudenheim JL, Genco RJ, LaMonte MJ, Millen AE, Hovey KM, Mai X (2016). Periodontal disease and breast cancer: prospective cohort study of postmenopausal women. Cancer Epidemiol Biomarkers Prev.

[ref40] Gao LG, Ni XY, Lin T, Fang M, Lin T (2016). Effect of 16-bit computed tomography imaging of metallic implants on dose distribution in radiotherapy. Chinese J Radiation Oncol.

[ref41] Gnant M (2011). Intravenous bisphosphonates for breast cancer: impact on patient outcomes and scientific concepts. Breast Dis.

[ref42] Guo L, Liu S, Zhang S, Chen Q, Zhang M, Quan P (2015). C-reactive protein and risk of breast cancer: A systematic review and meta-analysis. Sci Rep.

[ref43] Güven DC, Dizdar Ö, Akman AC, Berker E, Yekedüz E, Ceylan F (2019). Evaluation of cancer risk in patients with periodontal diseases. Turk J Med Sci.

[ref44] Han MA (2017). Oral health status and behavior among cancer survivors in Korea using nationwide survey. Int J Environ Res Public Health.

[ref45] Hao Y, Peng X, Zhou X-D, Cheng L (2019). Research progress on the relationship between periodontal disease and common malignancies. West China J Stomatol.

[ref46] Hartnett E (2015). Integrating oral health throughout cancer care. Clin J Oncol Nurs.

[ref47] Hegde R, Awan KH (2019). Effects of periodontal disease on systemic health. Dis Mon.

[ref48] Heikkilä P, But A, Sorsa T, Haukka J (2018). Periodontitis and cancer mortality: Register-based cohort study of 68,273 adults in 10-year follow-up. Int J Cancer.

[ref49] Hoare A, Soto C, Rojas-Celis V, Bravo D (2019). Chronic Inflammation as a link between periodontitis and carcinogenesis. Mediators Inflamm.

[ref50] Hoefert S, Grimm M, Sharghi F, Geist A, Krimmel M, Reinert S (2014). Atraumatic tooth extraction in patients taking bisphosphonates: a review of literature and experience with three cases. Oral Maxillofac Surg.

[ref51] Huang LJ (2013). The effect of health education on the prognosis of elderly breast cancer patients after surgery. Chinese J Women Child Health Res.

[ref52] Huang X, Jiang MP, Bao SN, Yin YM (2021). Interpretation of key points in the 2021 CSCO “Guidelines for Diagnosis and Treatment of Breast Cancer”. Chinese J Oncol Surg.

[ref53] Huang XH, Qi BT, Yang J, Liu Y, Sun WB (2021). Effects of mechanical adjacent surface plaque control measures on periodontal nonsurgical treatment: a systematic review. Int J Stomatol.

[ref54] Hujoel PP, Drangsholt M, Spiekerman C, Weiss NS (2003). An exploration of the periodontitis-cancer association. Ann Epidemiol.

[ref55] Iliescu AA, Perlea P, Earar K, Gheorghiu IM, Iliescu MG, Iliescu A (2020). Dynamics of periodontal tissue in menopause. Ginecologia Ro.

[ref56] Iwata H, Saji S, Ikeda M, Sakai T, Sawaki M, Shien T (2020). The Japanese Breast Cancer Society Clinical Practice Guidelines, 2018 edition: the tool for shared decision making between doctor and patient [published correction appears in Breast Cancer 2021 Jul;28(4)987]. Breast Cancer.

[ref57] Jardim LC, Flores PT, do Carmo Dos Santos Araújo M, Chiesa J, de Moraes CMB, Antoniazzi RP (2020). Oral health-related quality of life in breast cancer survivors. Support Care Cancer.

[ref58] Jia M, Wu Z, Vogtmann E, O’Brien KM, Weinberg CR, Sandler DP (2020). The association between periodontal disease and breast cancer in a prospective cohort study. Cancer Prev Res (Phila).

[ref59] Jin J, Guang M, Ogbuehi AC, Li S, Zhang K, Ma Y (2021). Shared molecular mechanisms between Alzheimer’s disease and periodontitis revealed by transcriptomic analysis. Biomed Res Int.

[ref60] Joseph S, Curtis MA (2021). Microbial transitions from health to disease. Periodontol 2000.

[ref61] Kamarajan P, Ateia I, Shin JM, Fenno JC, Le C, Zhan L (2020). Periodontal pathogens promote cancer aggressivity via TLR/MyD88 triggered activation of Integrin/FAK signaling that is therapeutically reversible by a probiotic bacteriocin. PLoS Pathog.

[ref62] Kamińska M, Ciszewski T, Łopacka-Szatan K, Miotła P, Starosławska E (2015). Breast cancer risk factors. Prz Menopauzalny.

[ref63] Kapila YL (2021). Oral health’s inextricable connection to systemic health: Special populations bring to bear multimodal relationships and factors connecting periodontal disease to systemic diseases and conditions. Periodontol 2000.

[ref64] Kemer Doğan ES, Kırzıoğlu FY, Doğan B, Fentoğlu Ö, Kale B, Çarsancaklı SA (2018). The role of menopause on the relationship between metabolic risk factors and periodontal disease via salivary oxidative parameters. J Periodontol.

[ref65] Khosla S, Burr D, Cauley J, Dempster DW, Ebeling PR, Felsenberg D (2007). Bisphosphonate-associated osteonecrosis of the jaw: report of a task force of the American Society for Bone and Mineral Research. J Bone Miner Res.

[ref66] Kizub DA, Miao J, Schubert MM, Paterson AHG, Clemons M, Dees EC (2021). Risk factors for bisphosphonate-associated osteonecrosis of the jaw in the prospective randomized trial of adjuvant bisphosphonates for early-stage breast cancer (SWOG 0307). Support Care Cancer.

[ref67] Koivisto L, Bi J, Häkkinen L, Larjava H (2018). Integrin αvβ6: Structure, function and role in health and disease. Int J Biochem Cell Biol.

[ref68] Krishna R, De Stefano JA (2016). Ultrasonic vs. hand instrumentation in periodontal therapy: clinical outcomes. Periodontol 2000.

[ref69] Kwon T, Lamster IB, Levin L (2021). Current concepts in the management of periodontitis. Int Dent J.

[ref70] Lai YH, Wu ZL (2012). Infection and anti-infection treatment of leukemia in children. J Applied Clinical Pediatr.

[ref71] Lesclous P, Abi Najm S, Carrel JP, Baroukh B, Lombardi T, Willi JP (2009). Bisphosphonate-associated osteonecrosis of the jaw: a key role of inflammation?. Bone.

[ref72] Li T, Mello-Thoms C, Brennan PC (2016). Descriptive epidemiology of breast cancer in China: incidence, mortality, survival and prevalence. Breast Cancer Res Treat.

[ref73] Li ZB, Li K (2019). Research progress on the mechanism of estrogen influencing periodontal tissue. J Oral Med Res.

[ref74] Lin JN, Liu Q (2020). Interpretation of the 2019 NCCN Breast Cancer Clinical Practice Guidelines Update: New Progress in Local Treatment of Breast Cancer. J Clin Surg.

[ref75] Luo L, Zheng W, Chen C, Sun S (2021). Searching for essential genes and drug discovery in breast cancer and periodontitis via text mining and bioinformatics analysis. Anticancer Drugs.

[ref76] Ma H, Zheng J, Li X (2020). Potential risk of certain cancers among patients with periodontitis: a supplementary meta-analysis of a large-scale population. Int J Med Sci.

[ref77] Mai X, LaMonte MJ, Hovey KM, Freudenheim JL, Andrews CA, Genco RJ (2016). Periodontal disease severity and cancer risk in postmenopausal women: the Buffalo OsteoPerio Study. Cancer Causes Control.

[ref78] Marwaha AK, Morris JA, Rigby RJ (2020). Hypothesis: bacterial induced inflammation disrupts the orderly progression of the stem cell hierarchy and has a role in the pathogenesis of breast cancer. Med Hypotheses.

[ref79] Maximov PY, Abderrahman B, Curpan RF, Hawsawi YM, Fan P, Jordan VC (2018). A unifying biology of sex steroid-induced apoptosis in prostate and breast cancers. Endocr Relat Cancer.

[ref80] Michaud DS, Lu J, Peacock-Villada AY, Barber JR, Joshu CE, Prizment AE (2018). Periodontal disease assessed using clinical dental measurements and cancer risk in the ARIC study. J Natl Cancer Inst.

[ref81] Napeñas JJ, Brennan MT, Coleman S, Kent ML, Noll J, Frenette G (2010). Molecular methodology to assess the impact of cancer chemotherapy on the oral bacterial flora: a pilot study. Oral Surg Oral Med Oral Pathol Oral Radiol Endod.

[ref82] Nicolatou-Galitis O, Schiødt M, Mendes RA, Ripamonti C, Hope S, Drudge-Coates L (2019). Medication-related osteonecrosis of the jaw: definition and best practice for prevention, diagnosis, and treatment. Oral Surg Oral Med Oral Pathol Oral Radiol.

[ref83] Nirola A, Batra P, Kaur J (2018). Ascendancy of sex hormones on periodontium during reproductive life cycle of women. J Int Clin Dent Res Org.

[ref84] Nwizu NN, Marshall JR, Moysich K, Genco RJ, Hovey KM, Mai X (2017). Periodontal disease and incident cancer risk among postmenopausal women: results from the Women’s Health Initiative Observational Cohort. Cancer Epidemiol Biomark Prev.

[ref85] Otto S, Schreyer C, Hafner S, Mast G, Ehrenfeld M, Stürzenbaum S (2012). Bisphosphonate-related osteonecrosis of the jaws – characteristics, risk factors, clinical features, localization and impact on oncological treatment. J Craniomaxillofac Surg.

[ref86] Pan YP (2017). [Treatment decision for periodontal patients with history of cancer].

[ref87] Parhi L, Alon-Maimon T, Sol A, Nejman D, Shhadeh A, Fainsod-Levi T (2020). Breast cancer colonization by Fusobacterium nucleatum accelerates tumor growth and metastatic progression. Nat Commun.

[ref88] Parida S, Sharma D (2019). The microbiome-estrogen connection and breast cancer risk. Cells.

[ref89] Parida S, Sharma D (2019). The power of small changes: Comprehensive analyses of microbial dysbiosis in breast cancer. Biochim Biophys Acta Rev Cancer.

[ref90] Parveen Rajpar DS, Ahmed Banglani DM, Kumar Punjabi DS, Priya D (2016). Dental floss; concept and use among the undergraduate dental students. Prof Med J.

[ref91] Pinton G, Moro L (2017). Expression and therapeutic significance of estrogen receptor β in malignant pleural mesothelioma. Future Sci OA.

[ref92] Poulias E, Melakopoulos I, Tosios K (2011). Metastatic breast carcinoma in the mandible presenting as a periodontal abscess: a case report. J Med Case Rep.

[ref93] Proença MA, Biselli JM, Succi M, Severino FE, Berardinelli GN, Caetano A (2018). Relationship between Fusobacterium nucleatum, inflammatory mediators and microRNAs in colorectal carcinogenesis. World J Gastroenterol.

[ref94] Professional Committee of Periodontology of the Chinese Stomatological Association (2017). The Chinese Expert Consensus on the Diagnostic Criteria of Severe Periodontitis and the Principles of Periodontal Disease Treatment in Special Populations. Chinese J Stomatol.

[ref95] Puspitadewi SR, Wulandari P, Masulili SLC, Auerkari E, Iskandar H, Yavuz I (2017). The relation of follicle stimulating hormone and estrogen to mandibular alveolar bone resorption in postmenopausal women. J Int Dent Med Res.

[ref96] Radaic A, Ganther S, Kamarajan P, Grandis J, Yom SS, Kapila YL (2021). Paradigm shift in the pathogenesis and treatment of oral cancer and other cancers focused on the oralome and antimicrobial-based therapeutics. Periodontol 2000.

[ref97] Regev E, Lustmann J, Nashef R (2008). Atraumatic teeth extraction in bisphosphonate-treated patients. J Oral Maxillofac Surg.

[ref98] Rezaei M, Khoshay A, Amirifard N, Goli A, Abdi A (2021). Comparison of the effect of alpha and hydrocortisone ointments on prevention of acute skin complications due to radiotherapy in breast cancer patients. J Skin Cancer.

[ref99] Rizzoli R, Body JJ, DeCensi A, Reginster JY, Piscitelli P, Brandi ML (2012). Guidance for the prevention of bone loss and fractures in postmenopausal women treated with aromatase inhibitors for breast cancer: an ESCEO position paper [published correction appears in Osteoporos Int 2012 Nov;23: 2577. De Censi, A [corrected to DeCensi, A]. Osteoporos Int.

[ref100] Robinson JL, Johnson PM, Kister K, Yin MT, Chen J, Wadhwa S (2020). Estrogen signaling impacts temporomandibular joint and periodontal disease pathology. Odontol.

[ref101] Saito H, Watanabe Y, Sato K, Ikawa H, Yoshida Y, Katakura A (2014). Effects of professional oral health care on reducing the risk of chemotherapy-induced oral mucositis. Support Care Cancer.

[ref102] Schmidt A, Ernstmann N, Wesselmann S, Pfaff H, Wirtz M, Kowalski C (2016). After initial treatment for primary breast cancer: information needs, health literacy, and the role of health care workers. Support Care Cancer.

[ref103] Sfreddo CS, Maier J, De David SC, Susin C, Moreira CHC (2017). Periodontitis and breast cancer: A case-control study. Community Dent Oral Epidemiol.

[ref104] Shao J, Wu L, Leng WD, Fang C, Zhu YJ, Jin YH (2018). Periodontal disease and breast cancer: a meta-analysis of 1,73,162 participants. Front Oncol.

[ref105] Shao W, Fujiwara N, Mouri Y, Kisoda S, Yoshida K, Yoshida K (2021). Conversion from epithelial to partial-EMT phenotype by Fusobacterium nucleatum infection promotes invasion of oral cancer cells. Sci Rep.

[ref106] Shapiro LF, Freeman K (2014). The relationship between estrogen, estrogen receptors and periodontal disease in adult women. J Mich Dent Assoc.

[ref107] Sheehy EM, Lehane E, Quinn E, Livingstone V, Redmond HP, Corrigan MA (2018). Information needs of patients with breast cancer at years one, three, and five after diagnosis. Clin Breast Cancer.

[ref108] Siegel RL, Miller KD, Jemal A (2020). Cancer statistics, 2020. CA Cancer J Clin.

[ref109] Siqueira TC, Frágoas SP, Pelegrini A, de Oliveira AR, da Luz CM (2021). Factors associated with upper limb dysfunction in breast cancer survivors. Support Care Cancer.

[ref110] Söder B, Yakob M, Meurman JH, Andersson LC, Klinge B, Söder PÖ (2011). Periodontal disease may associate with breast cancer. Breast Cancer Res Treat.

[ref111] Soory M, Tilakaratne A (2003). Modulation of androgen metabolism by phenytoin, oestradiol and tamoxifen in human gingival fibroblasts. J Clin Periodontol.

[ref112] Soutome S, Otsuru M, Kawashita Y, Funahara M, Ukai T, Saito T (2021). Effect of cancer treatment on the worsening of periodontal disease and dental caries: a preliminary, retrospective study. Oral Health Prev Dent.

[ref113] Stein JM, Yekta-Michael SS, Schittenhelm F, Reichert S, Kupietz D, Dommisch H (2021). Comparison of three full-mouth concepts for the non-surgical treatment of stage III and IV periodontitis: A randomized controlled trial. J Clin Periodontol.

[ref114] Taichman LS, Gomez G, Inglehart MR (2014). Oral health-related complications of breast cancer treatment: assessing dental hygienists’ knowledge and professional practice. J Dent Hyg.

[ref115] Taichman LS, Inglehart MR, Giannobile WV, Braun T, Kolenic G, Van Poznak C (2015). Periodontal health in women with early-stage postmenopausal breast cancer newly on aromatase inhibitors: a pilot study. J Periodontol.

[ref116] Taichman LS, Van Poznak CH, Inglehart MR (2018). Oral health-related concerns, behavior, and communication with health care providers of patients with breast cancer: impact of different treatments. Spec Care Dentist.

[ref117] Taichman LS, Van Poznak CH, Inglehart MR (2016). Self-reported oral health and quality of life of postmenopausal breast cancer survivors on aromatase inhibitors and women without cancer diagnoses: a longitudinal analysis. Support Care Cancer.

[ref118] Taiwan BJ (2018). Oral health status and prevention and control strategies of Chinese residents, interpretation of the results of the Fourth National Oral Health Epidemiological Survey. In: The 13th National Geriatric Stomatology Annual Conference.

[ref119] Travis RC, Key TJ (2003). Oestrogen exposure and breast cancer risk. Breast Cancer Res.

[ref120] Urbaniak C, Cummins J, Brackstone M, Macklaim JM, Gloor GB, Baban CK (2014). Microbiota of human breast tissue. Appl Environ Microbiol.

[ref121] Ustaoğlu G, Göller Bulut D, Üyetürk Ü, Uysal Ö (2021). Evaluation of periodontal health in breast cancer patients undergoing tamoxifen or aromatase inhibitors drugs therapy: A cross-sectional study. Spec Care Dentist.

[ref122] Valente NA, Andreana S (2017). A combined treatment for a case of peri-implant bisphosphonate-related osteonecrosis of the jaw. J Int Acad Periodontol.

[ref123] Vargas-Villafuerte KR, Dantas FT, Messora MR, Novaes AB, Grisi MF, Taba M (2016). Preliminary results of non-surgical periodontal treatment in patients with breast cancer undergoing chemotherapy. J Periodontol.

[ref124] Vaz MMOLL, de Jesus Guirro RR, Carrara HHA, Montezuma T, Perez CS, de Oliveira Guirro EC (2017). Alteration of blood circulation in the upper limb before and after surgery for breast cancer associated with axillary lymph node dissection or sentinel lymph node biopsy. Lymphat Res Biol.

[ref125] Vieira AT, Castelo PM, Ribeiro DA, Ferreira CM (2017). Influence of oral and gut microbiota in the health of menopausal women. Front Microbiol.

[ref126] Villafuerte KRV, Dantas FT, Taba M, Messora M, Candido Dos Reis FJ, Carrara HHA (2021). Effects of non-surgical periodontal therapy on the cytokine profile in gingival crevicular fluid of breast cancer patients with periodontitis undergoing chemotherapy. Support Care Cancer.

[ref127] Vinayak S, Davidson NE (2019). Extending adjuvant endocrine therapy in breast cancer: who, what, why?. Oncol (Williston Park).

[ref128] Vozza I, Caldarazzo V, Polimeni A, Ottolenghi L (2015). Periodontal disease and cancer patients undergoing chemotherapy. Int Dent J.

[ref129] Wilcock P, Webster RM (2021). The breast cancer drug market. Nat Rev Drug Discov.

[ref130] Willershausen I, Schmidtmann I, Azaripour A, Kledtke J, Willershausen B, Hasenburg A (2019). Association between breast cancer chemotherapy, oral health and chronic dental infections: a pilot study. Odontology.

[ref131] Wong HM (2014). Oral complications and management strategies for patients undergoing cancer therapy. Sci World J.

[ref132] Wu K, Zhang H, Fu Y, Zhu Y, Kong L, Chen L (2018). TLR4/MyD88 signaling determines the metastatic potential of breast cancer cells. Mol Med Rep.

[ref133] Wu X, Li F, Dang L, Liang C, Lu A, Zhang G (2020). RANKL/RANK system-based mechanism for breast cancer bone metastasis and related therapeutic strategies. Front Cell Dev Biol.

[ref134] Zhang JY (2018). The impact of radiotherapy and chemotherapy on periodontium and periodontal management for patients with head and neck malignancy. J Dent Endodon.

[ref135] Zhang Y, Mao X, Yu X, Huang X, He W, Yang H (2022). Bone mineral density and risk of breast cancer: A cohort study and Mendelian randomization analysis. Cancer.

[ref136] Zhang Y, Qiao D, Chen R, Zhu F, Gong J, Yan F (2021). The association between periodontitis and inflammatory bowel disease: a systematic review and meta-analysis. Biomed Res Int.

[ref137] Zhang YN, Liu LR (2013). Analysis of relationship between doctors and patients and hospital legal system culture: from Szasz & Hollender’s model of doctor-patient relationship as the theoretical perspective. Chin Hosp Mgmt.

